# A Reply to "an Old Practitioner, on the Frauds Practised by Persons to Gain Admission to the Medical Profession, and the Necessity of Counteracting Them."*

**Published:** 1821-12

**Authors:** J. Charleton Yeatman

**Affiliations:** Surgeon Extraordinary to his Royal Highness the Duke of Gloucester; Member of the Royal College of Surgeons; and late on the Hospital Staff in the Mediterranean.


### *A Reply* to "an Old Practitioner, on the Frauds practised by Persons to gain admission to the Medical Profession, and the Necessity of counteracting them."* By J. CHARLETON YEATMAN, Surgeon Extraordinary to his Royal Highness the Duke of Gloucester; Member of the Royal College of Surgeons; and late on the Hospital Staff in the Mediterranean.

Justitiæ partes sunt non violare homines: verecundiæ non offendere.—TULL.

IN the Journal for July appears a communication, consisting of two paragraphs, with the above high-sounding and imposing title! It contains some assertions and insinuations which are evidently intended to injure some particular member of the profession, under the specious pretence of watching over its best interest.

"I live," says this anonymous writer, "in the neighbourhood of a practitioner who has taken upon himself to qualify pupils for medical practice in the short space of a few months." Now, if this be true, the honour of the profession, and a duty which every member of it owes to the public, loudly call on the writer to substantiate the charge: nor has any man, thus *worthily engaged*, aught to fear in SUBSCRIBING HIS NAME. But what will my readers say when I inform them, that the above communication has been read in this town by gentlemen who are not in the habit of perusing medical publications, and who can hardly be supposed to have read the identical Number of that Journal in which the communication appeared, *unless* it had been previously pointed out by some individual, with a view to injure a particular member of the profession in this town or neighbourhood!

---

* This communication would have appeared in the Journal for September last, but for the communication between the late Editor (Dr. Hutchinson) and myself, relative to the *real name** of "an Old Practitioner."

* The *real name* of "an Old Practitioner," we have the authority of Dr. Hutchinson to state, is only known to himself.—A. B. G.

Trusting I shall say enough to convince the reader of the propriety of *my* taking some notice of the paragraphs in question, I shall proceed to state further, that those individuals, in Frome, who have seen them, are of opinion that the writer intended them (however unjustly) to apply to myself: first, because one of my pupils was only to remain with me *one year;* secondly, because the parenthetical expression, "tired of his own pursuits," alludes (however absurdly) to a lieutenant R.N., who, during a peace which is not likely to be disturbed, and favoured by the genius of medicine, has, very laudably, directed his thoughts to the acquisition of another profession; thirdly, because the communication in point is considered to allude to one who is in the habit of giving lectures; and neither my neighbours nor myself are aware that any other practitioner in the county of Somerset, whence the author dates his communication, is thus professionally engaged.

I will now correct some of those errors into which the writer has fallen.

The anonymous author has affirmed, that "the law precludes all persons from admission into the chartered bodies of surgeons and apothecaries, but such as have *(bona fide)* served a regular term of five years as apprentices."

Now, as to the *law* on this point, the late Editor of this Journal has shown the author's mistake, and explained it sufficiently. I will therefore only add, for the writer's information, with respect to candidates for examination at the College of Surgeons, of London, that one of the qualifications is that of having been ENGAGED five years, at least, in the acquisition of professional knowledge;* and further, that "candidates, under extraordinary circumstances of professional education, not literally corresponding with the foregoing rules, but deemed by the Court in effect equivalent thereto, may be admitted to examination."†

The wisdom of the College is here manifest: they did not forget that some of the brightest ornaments of the profession were pupils *not bound by indenture;* and, lest men should be prevented from becoming members of the College who might be engaged in the study of medicine later in life than usual,—men, both talented and industrious, too old to serve as *apprentices*, and yet peculiarly and advantageously situated,—the College do not insist on indentures as a *sine quâ non,* and will admit to examination, for a diploma, "candidates, under extraordinary circumstances of professional education," &c.‡

---

* See Rules relating to Candidates, signed, by order, Edward Belfour Secretary. † Ibid.

‡ The present Editor received a diploma of member of the College of Surgeons, which enabled him to serve in the Royal Navy, without ever having been an apprentice, by simply exhibiting, on the day of examination, the usual certificates of having received a regular medical education.—A. B. G.

It will be proper in this place to notice, briefly, the system which I adopt with regard to pupils, and which the anonymous writer has attempted, it is supposed, to disparage. I educate two or three gentlemen for the profession, either as pupils or apprentices: the former engage with me for one year or more, to whom I grant certificates of attendance to my lectures on anatomy, with remarks on the principles and practice of physic, surgery, and midwifery; of having been instructed in pharmacy; and attended, with me, the sick and hurt poor in this populous manufacturing town. The latter are apprenticed for five years, with liberty to leave me in four, or to remain the full period of the term, as shall be deemed most advisable. Both pupils and apprentices then proceed, as usual, to one of the great medical schools, either to complete their education as surgeons or to graduate as physicians.

"An Old Practitioner" asserts, with all the gravity of one who is recording an important truth, (and it is really lamentable to find so much want of good sense in a single page,) "that no person should be allowed to practise, whose *mind has not been stored by long and patient observation;*" as if that could be attained, during an apprenticeship, which is solely the result of *great* experience and talent!

But, to conclude. The great utility, to medical pupils, of a system of education, like that which I have noticed, must be obvious to all who are not wilfully blind to a subject of this nature. Surely, there can be no necessity for relating the advantages resulting from a systematic course of professional instruction, a parallel to which might be found in the law department, as regards barristers before, as well as after, they proceed to the Temple, under special pleaders; and in the church, by young divines studying under private tutors, before, as well as after, they enter at the University,—*not to supersede the necessity of going to the University and Temple, but to give effect to their studies whilst there;*—a preparatory system, an undertaking of a vow, as it were, *solvendum in futuro.*

Si quid novisti rectius istis,

Candidus imperti; si non, his utere mecum.—Hor. Ep.

---

In the 31st volume of the Journal, I have stated a case of recovery from an ounce of laudanum which had been retained in the stomach three hours; and, for the reasons which in a subsequent paper (in the 32d volume,) I advanced, I endeavoured to show that, instead of death being immediately caused, in similar cases, by "congestion or stagnation of the blood in the vessels of the brain and of the lungs," (for which bleeding is advised by the celebrated Cullen and others,) opium kills by destroying the functions of the brain and nerves, in consequence of its sedative effects on the stomach, par vagum, and great sympathetic nerves. On this principle, I adopted and recommended the prompt use of powerful emetics, vegetable acids, and strong stimuli; deprecating the early use of the lancet, and illustrating my position by a case of death, in which an American physician opened the temporal artery the moment re-action had been established by the use of the above remedies. I noticed the paucity of cases wherein opium or laudanum is swallowed with a view to destroy life, and begged those of your readers who may have met with any to narrate them, with their treatment and event. I again embrace the opportunity of calling their attention to this important subject, and I beg to observe, that those cases which may have occurred to them, the details of which may not have been sufficiently preserved to admit of separate publication, I should most thankfully receive, by letter; since even the recital of the quantum of opium taken into the stomach,—the length of time it remained there,—the leading symptoms,—the remedies employed,—and the event of each case, may throw considerable light on a subject, which, since the publication of the papers alluded to I have not lost sight of.

---

The child, Sarah Slade, whose foot I amputated at the instep, (see 37th volume,) still walks with a firm tread, and without any visible deformity. Perhaps "an Old Practitioner" can inform me that, in a case of this kind, *nature makes a better stump than the surgeon*. The same *great medical authority* may also be good enough to show that *it is quite impossible to detect a scirrhous pylorus*, and indeed a tumor at that extremity of the stomach, *by the touch, in an extremely emaciated person;* and may illumine the medical world with his remarks *in favour of sutures in severely contused lacerated wounds of the extremities!*

*Frome, Somerset; Oct. 3d*, 1821.

